# The Mini-International Neuropsychiatric Interview is useful and well accepted as part of the clinical assessment for depression and anxiety in primary care: a mixed-methods study

**DOI:** 10.1186/s12875-017-0674-5

**Published:** 2018-01-24

**Authors:** Agneta Pettersson, Sonja Modin, Rolf Wahlström, Sandra af Winklerfelt Hammarberg, Ingvar Krakau

**Affiliations:** 1 Medical Management Centre, Department of Learning, Informatics, Management and Ethics, Karolinska Institutet, Tomtebodavägen 18 A, SE-171 77 Stockholm, Sweden; 2 Swedish Agency for Health Technology Assessment and Social Assessment, SE-102 33 Stockholm, Sweden; 3 Division of Family Medicine, Department of Neurobiology, Care Sciences and Society, Karolinska Institutet, Alfred Nobels allé 23 D2, SE-141 83 Huddinge, Sweden; 4 Department of Public Health Sciences, Karolinska Institutet, Tomtebodavägen 18 A, SE-171 77 Stockholm, Sweden; 5 Department for Neurobiology, Care Sciences and Society, Karolinska Institutet, Alfred Nobels allé 23 D2, SE 141 83 Huddinge, Sweden; 6 Department of Medicine, Clinical Epidemiology Unit T2, 14, Karolinska Institutet, SE-171 77 Stockholm, Sweden

**Keywords:** Depression, Anxiety, Differential diagnosis, Qualitative content analysis, MINI, Acceptability

## Abstract

**Background:**

Psychiatric complaints are common among primary care patients, with depression and anxiety being the most frequent. Diagnosis of anxiety and depression can be difficult, potentially leading to over- as well as under-diagnosis. The diagnostic process can be facilitated by incorporating structured interviews as part of the assessment. One such instrument, the Mini-International Neuropsychiatric Interview (MINI), has been established and accepted in psychiatric care. The purpose of this study was to explore the experiences and perceptions of the paper-and-pen version of MINI version 6.0 among patients and staff in primary care centers in Sweden.

**Methods:**

The MINI was introduced at three primary care centers and was conducted by either therapists or general practitioners. Patients presented with symptoms that could suggest depression or anxiety disorders. The duration of the interview was recorded. The experiences and perceptions of 125 patients and their interviewers were collected using a structured questionnaire. Global satisfaction was measured with a visual-analog scale (0–100). Semi-structured interviews were conducted with 24 patients and three therapists, and focus groups were held with 17 general practitioners. Qualitative content analysis was used for the interviews and focus groups. The findings across the groups were triangulated with results from the questionnaires.

**Results:**

The median global satisfaction with the MINI was 80 for patients and 86 for interviewers. General practitioners appreciated that the MINI identified comorbidities, as one-third of the patients had at least two psychiatric diagnoses. The MINI helped general practitioners attain a more accurate diagnosis. Patients appreciated that the MINI helped them recognize and verbalize their problems and did not find it intrusive. Patients and interviewers had mixed experiences with the yes-no format of the MINI, and the risk of subjective interpretations was acknowledged. Patients, general practitioners and therapists stated that the MINI contributed to appropriate treatment. The MINI assessment lasted 26 min on average (range 12 to 60 min).

**Conclusions:**

The paper-and-pen version of the MINI could be useful in primary care as part of the clinical assessment of patients with problems suggestive of depression or anxiety disorders. The MINI was well accepted by patients, general practitioners and therapists.

**Electronic supplementary material:**

The online version of this article (10.1186/s12875-017-0674-5) contains supplementary material, which is available to authorized users.

## Background

Psychiatric problems are common among patients in primary care [1], with depressive and anxiety disorders being the most frequent diagnoses [1–5]. Comorbidity of depressive and anxiety disorders is also common [1, 3, 6]. A correct diagnosis of any psychiatric problem is vital to enabling optimal treatment but may be difficult to establish, e.g., owing to somatic and psychiatric comorbidities [7, 8].

A governmental health technology assessment report on instruments to support the diagnosis of mood disorders [9] inspired the present study, which initially focused on depression. A previous systematic review concluded that half of patients with depression were not recognized [10]. Furthermore, some studies on depression have shown that a substantial proportion of patients remain undiagnosed after several visits to a general practitioner (GP) [4, 11]. However, other studies have indicated that awareness of depression may result in a diagnosis even when the patient has only subthreshold symptoms [12, 13].

Attempts have been made to help GPs improve their skills in diagnosing depression, including by providing training in consultation techniques [14–17] and through the use of short questionnaires or structured interviews to complement the consultation [18–20]. However, a systematic review concluded that few of these instruments had acceptable sensitivity and specificity for depression [21]. One exception was the Mini-International Neuropsychiatric Interview (MINI) instrument, which is a structured interview; at the time of the study, the MINI 6.0, based on the DSM-IV, was available. The MINI comprises modules for 17 psychiatric diagnoses. Questions are phrased to allow only “yes” or “no” answers. Examples are provided to facilitate responses and should be read word for word. The MINI is available in both a paper-and-pen and a web-based version.

For structured interviews to have utility in practice, they must also be acceptable to patients and staff. However, no studies on primary care patients’ perspectives of the MINI could be retrieved (August 2016). One study, from Brazil, found that general practitioners (GPs) who used the MINI were satisfied with the interview [22]. Two studies, from Italy and Norway, showed that the MINI was well accepted by patients and interviewers in psychiatric care [23, 24]. In Sweden, few GPs use the MINI. According to a postal survey in 2011 of 300 randomly chosen Swedish GPs (42% response rate), only five of them (4%) had used the MINI [9].

As the MINI is not limited to depression and as there is significant co-morbidity of depression and anxiety, the focus of the present study shifted toward evaluating the use of the MINI for patients with symptoms suggestive of any of these diagnoses. This expansion better mirrored the primary care patient population. The MINI has shown high accuracy for depression in psychiatric as well as in primary care [9, 22, 25]. There is less information about its accuracy for other diagnoses, but it is acceptable for panic disorder and generalized anxiety disorder in psychiatric and primary care settings [22, 25, 26]. The accuracy of the MINI for agoraphobia and social anxiety disorders has been found to be acceptable when measured in psychiatric settings [25, 26].

The purpose of the present study was to explore the experiences of and perceptions of the paper-and-pen version of the MINI among staff and patients for patients with problems suggestive of depression or anxiety disorders in Swedish primary care centers (PCCs).

## Methods

This was a pragmatic mixed-methods study that did not provide any financial support to the participating PCCs. The study was designed to minimize extra administrative work by participating staff.

### Setting and participants

This study was carried out in Stockholm County between February 2014 and March 2015. After establishing the study with relevant decision-makers in the county council and political leadership, PCCs were recruited through personal contacts. Seven PCCs were approached, and three agreed to participate, here called PCC1, PCC2 and PCC3. The PCCs had two introductory meetings, 1 h each, led by three of the authors (AP, IK and SW). The MINI and supportive evidence were presented during the first meeting, whereas the study protocol was addressed in the second meeting. Some characteristics of the PCCs are described in Table 1. At PCC1, the MINI was implemented as part of the study. Patients were referred to a medical social worker experienced in Cognitive Behavior Therapy who conducted the MINI interview. The interviewer received a full-day training on the use of the MINI led by one of the authors (SW). At PCC2 and PPC3, the MINI was already in routine use, and the interview was conducted by certified psychologists or by GPs themselves. The psychologists and the medical social worker are referred to as therapists in this article.

**Table 1:** Characteristics of participating primary care centers (PCC)

PCC ID	Location, listed patients (n); CNI^a^	Number parti-cipating GPs (number employed)	MINI interviewer	Procedure
1	Suburb, 18,000 patients; CNI = 1.26	9 (15)	1 medical social worker after referral from a GP	MINI results fed back to the GP for decision on further management
2	Suburb, 21,000 patients; CNI = 0.93	≥8 (14)	Psychologists after referral from a GP **or** the GPs themselves	Psychologists made a full assessment including MINI and initiated CBT if relevant **or** the GPs initiated treatment.
3	Central Stockholm, 10,000 patients; CNI = 0.72	1 (3)	The GP	The GP initiated treatment

^a^*CNI* Care Need Index [45] a measure of psychosocial burden, where higher values indicate larger problems; average CNI = 1.0; *GP* general practitioner

Eligible patients met at least one of the following criteria: an ongoing episode of major depression that had not responded adequately to treatment after 2 months; a new episode of a mental health problem; somatic symptoms in which depression could be a differential diagnosis; and frequent attendance at the PCC as perceived by the GP [27]. Patients had to be 18 years or older, sufficiently capable in the Swedish language to participate in the interview and lacking cognitive deficiencies. Patients who needed immediate treatment were excluded. Only patients who consented to completing a questionnaire after the MINI were included in the study. The MINI assessment was performed by three employed therapists (1 at PCC1 and 2 at PCC2) and four GPs (three at PCC2 and one at PCC3). The number of contract therapists who contributed ten questionnaires at PCC2 was not retrieved.

### Data collection

The perceptions and experiences of patients and interviewers were gathered from a structured questionnaire on acceptance. To obtain a deeper understanding, the questionnaires were supplemented with semi-structured interviews or focus groups.

#### Questionnaires

The questionnaire addressed the emotional and mental aspects of using the MINI, with one version for the patients and another for the interviewers (Additional file 1). The questionnaires were developed and validated in Germany for a study [28] on the acceptance of another structured interview, Diagnostisches Interview bei Psychischen Störungen (DIPS for the DSM-IV-TR), and were used with the permission of the research group. The questionnaires were translated into Swedish by one of the authors (AP) and back-translated by a teacher working in Sweden whose native language was German. The accuracy of the back-translated text was verified by one of the leading researchers working with the DIPS. In short, the questionnaire comprised ten Likert-type statements scored from 0 = do not agree at all to 3 = agree fully. The respondents also estimated their global satisfaction with the MINI on a Visual Analog scale (VAS, 0 to 100). In addition to completing the questionnaire, the patients shared their reasons for visiting the PCC. The interviewers also recorded the time required for the MINI assessment, including the administration and scoring, the gender and age of the patients and the results of the assessment. The questionnaires were completed directly after the MINI assessment.

#### Interviews and focus group discussions

The interviews and focus group discussions were based on topic guides that included probing questions when necessary (Additional file 1). They were audio-recorded and supplemented with field notes.

Patients were interviewed individually by one of the authors (AP). A purposeful sample was recruited with the goal of obtaining an even distribution between PCCs and a wide variation in gender, age and ethnicity. All patients who completed the questionnaire were asked to participate in a face-to-face interview. Those who volunteered were contacted, and the interviews were performed concurrently. The participants could choose the time and location of the interview (home, the interviewer’s office or the PCC). The interviews took an average of 25 min, and the participants were compensated with a voucher for a cinema ticket (value of 12 euro). Out of 125 patients, 49 signed up for an interview. Nine (four men) did not consent, and 16 women aged 25–60 years were not contacted. Thus, 24 persons, six men and 18 women, were interviewed; four were younger than 25 years, and five were older than 60 years. Six persons were first- or second-generation immigrants. Transcripts were not returned to the participants for verification, and feedback of results was not provided.

The first author (AP) interviewed the three therapists at their respective PCCs after study completion. All GPs at PCC1 and PCC2 were invited to a focus group discussion. Focus groups were chosen because the interactions between participants can facilitate the emergence of different aspects of interest, which is especially fruitful when there is scarce information about the phenomenon beforehand [29]. Two focus groups were held at PCC1 (4 and 5 participants), and one at PCC2 (8 participants). Three of the GPs were men, and 14 were women. Three were receiving specialist training, and seven had worked as GPs for more than 20 years. The GP at PCC3 was one of the authors and did not participate in a focus group. The focus groups were moderated by AP and IK, who ensured that all participants shared their experiences. The average duration of the group discussions was 45 min. The therapists and GPs did not receive compensation other than a light meal. Neither AP nor IK knew the GPs and therapists beforehand. Interviewees were informed that the study was part of the interviewer’s thesis and was of interest to the Stockholm County Council.

### Data analysis

Data from the acceptance questionnaires were managed in Excel 2013. The VAS scores regarding global satisfaction with the MINI assessment were estimated with a ruler. The scores had a right-skewed distribution; therefore; the medians and interquartile range (IQR) were calculated. For the ten items on the acceptance questionnaires, the proportion of participants who almost or fully agreed with the statement (rated 2 or 3) was calculated.

The time required for the assessments was analyzed as the mean (SD) number of minutes.

The interviews and focus group discussions were transcribed verbatim by a secretary who was external to the study, and the transcripts were validated against the recordings by AP. The analytical approach was based on qualitative content analysis [30, 31] and was inductive, as research on this phenomenon is scarce [31]. Coding was performed separately for patients, interviewers and GPs. Excerpts regarding the structure and questions of the MINI from the GPs who conducted the MINI were analyzed together with the other interviewers’ statements.

The preliminary extraction, condensing, and coding of meaning units was performed by AP and verified by SM. The subcategories and categories were first defined independently by SM and AP and were then confirmed by consensus. In the next step, categories across participant groups were grouped under common main categories. The entire process was conducted iteratively, continuously considering other possibilities and involving all authors. Finally, the items from the questionnaires were mapped onto the main categories for integration of the data sets. Data were managed in Word 2013 and Excel 2013. The results were presented at a meeting with GPs at PCC1, who expressed that the findings were in line with their experiences.

## Results

In total, 125 patients participated completed the questionnaire (Table 2). Of these, 22% had no MINI diagnosis, 47% had one diagnosis, and 31% fulfilled the criteria for at least two diagnoses. At PCC1, 67 patients fulfilled the inclusion criteria. Of these, five declined the MINI assessment, and another seven did not consent to the questionnaire. All patients who fulfilled the inclusion criteria at PCC3 consented to the study, but one did not complete the questionnaire. Drop-out data for the patients were not reported by PCC2. The interviewers at the three PCCs completed 115 questionnaires related to the participating patient; ten questionnaires were missing from PCC2.

**Table 2:** Characteristics of responding patients (*n* = 125), reasons for visits and MINI diagnosis

Characteristics	PCC1	PCC2	PCC3	Total
Number of patients	55	54	16	125
Number female patients	39 (72%)	45 (83%)	13 (81%)	97 (78%)
Age distribution^a^				
< 25 years	5	4	4	13 (12%)
25–60 years	41	31	12	84 (78%)
> 60 years	9	5	0	14 (13%)
Main reason to visit the PCC				
Depressive symptoms	16	18	7	41 (33%)
Anxiety	6	9	4	19 (15%)
Stress, tired, sleep problems	9	6	0	15 (12%)
Other psychiatric complaints	9	14	2	15 (20%)
Somatic complaints	12	3	2	17 (14%)
Not answered	3	4	1	8 (6%)
MINI diagnosis^b^				
Depression only	10	5	7	22 (18%)
One anxiety disorder only	11	21	0	32 (27%)
More than one anxiety disorder	3	2	0	5 (4%)
Both depression and anxiety disorder	11	3	7	21 (18%)
Depression or anxiety or both with other MINI diagnoses	6	3	2	11 (9%)
Other MINI diagnoses	1	2	9	3 (2%)
None	13	13	0	26 (22%)

^a^age reported for *n* = 40 at PCC2; ^b^ MINI diagnoses reported for *n* = 49 patients at PCC2

The experiences of patients, interviewers and referring GPs expressed in the interviews and focus groups centered around six main categories: Perceived strengths of the MINI; Perceived advantages of using the MINI for GPs; Perceived advantages of using the MINI for patients; Perceived weaknesses of the MINI; The duration of the MINI as a potential concern; and The MINI is an additional tool and a personal contact with the interviewer is important. An example coding scheme is shown in Additional file 2.

All items in the acceptance questionnaires corresponded to one of the main categories, with the exception of three items from the interviewer questionnaire. These items concerned their satisfaction with the assessment. The interviewers perceived that they felt competent and did not make any mistakes during the MINI. This issue was not discussed at all during the interviews and is not described further. Table 3 summarizes the main categories, their underlying categories and the corresponding items from the acceptance questionnaires (Table 3).

**Table 3:** Summary of findings for experiences and perceptions of using the MINI

Main category	PATIENTS category	INTERVIEWERS category	REFERRING GPs category	Corresponding item in the questionnaires
Perceived strengths of the MINI	Structured format and detailed questions that often capture the problem			P4, P10, I2
	The MINI does not evoke negative emotions		MINI does not evoke emotions	P3, P5, I8
Perceived advantages of using the MINI for GPs	More accurate diagnoses facilitate the work of the GP			
			A useful standard test for deeper investigations and selected patients	
Perceived advantages of using the MINI for patients	New insight into the problem			P2, P9, I5
	The MINI is mostly meaningful			P1, P6, I10
	The MINI may lead to better treatment			
Perceived weaknesses of the MINI	It is a constraint to only answer yes and no			
	Some questions are problematic, and some common problems are not covered			
	The results of the MINI may be biased			P8, I7
The duration of the MINI as a potential concern	The duration was acceptable	The MINI most often takes a short period of time		Time recording
		It could be problematic to fit the MINI into the GP consultation scheme		
The MINI is an additional tool and a personal contact with the interviewer is important	The MINI is just one part of the diagnostic procedure and its role must be explained			P7, I6, I9
	The personal contact is important for most patients			

Categories that are similar across the participant groups, patients, interviewers (therapists or GPs) and referring GPs are placed on the same row; empty space indicates that the subject was not discussed

The results of the questionnaires are shown in Table 4. They largely mirrored the findings of the interviews and focus groups. The overall satisfaction with the MINI as measured in the questionnaires was high. The median satisfaction among patients (*n* = 124) was 80 (IQR 64 to 92), with a range from 0 to 100. For the interviewers (*n* = 115 assessments), the median satisfaction was 86 (IQR 75 to 95), with a range from 25 to 100. In the interviews, patients and interviewers stated that the MINI was a good test.

**Table 4:** Results from the acceptance questionnaires

Patient questionnaire (*P*) (*n* = 125)		Interviewer questionnaire (*I*) (*n* = 115)	
Item (no.)	Percentage agreeing fully or almost fully	Item (no.)	Percentage agreeing fully or almost fully
The procedure was helpful (P1)	68	–	
I feel unclear about the results of MINI (P2)	23		
I felt interrogated (P3)	7		
Too many questions (P4)	6		
It was exhausting (P5)	4	It was exhausting (I8)	7
I felt taken seriously (P6)	93	I was responsive to the patient (I10)	85
A positive relationship with the interviewer was established (P7)	93	A positive relationship with the patient was established (I6)	87
		The patient was cooperative (I9)	96
I did not tell everything (P8)	16	The patient did not report everything (I7)	6
I understood my problems better (P9)	33	The patient only realizes some dimensions of her problem (I5)	1
It was enough detail for the interviewer (P10)	77	Difficulties to capture all relevant information (I2)	19
–		I conducted the interview as well as I could (I1)	100
–		I felt competent (I3)	88
–		I made mistakes (I4)	1

The table shows the proportions of questionnaires from patients and interviewers where the respondents agreed fully or almost fully to the items. Items that correspond to each other in the patient and interviewer questionnaires are placed on the same row

### Perceived strengths of the MINI

#### Structured format and detailed questions that often capture the problem

According to the questionnaires, most patients felt that the MINI had sufficiently detailed questions for the interviewer to understand the patient (Table 4). Less than 5 % perceived that there were too many questions (Table 4). The interviewers reported that they seldom encountered difficulties capturing all relevant information with the MINI (Table 4).

The standardized format also offered advantages. Patients appreciated that the questions were the same for everyone. One reason this consistency was appreciated was that it helped them realize that others could have the same problems and that they were not “odd or weird” (Patient 23). Interviewers agreed:

“The MINI plays down the situation for those who have a diagnosis: Apparently, there is a paper stating that you could have obsessions, so [obsessions] seem to be reasonably frequent” (GP3, PCC2).

Patients and interviewers also stated that the questions were detailed and most often easy to understand. However, the experiences with the examples included in the MINI were mixed. Some patients appreciated them because they increased their understanding of the item, whereas the examples were not helpful for others.

Some patients felt that it was easier to respond “yes” or “no” than to have to describe their situation and symptoms in own words. Thus, the MINI could facilitate the conversation for patients who were too tired to talk, had difficulties explaining how they felt or easily lost the thread of conversation while talking:

“…sometimes it feels as if you are only talking in circles, and you realize that you don’t understand where you are. This can be tough because then you wonder, ‘what was the purpose of this? Why did we talk about this?’” (Patient 23).

Patients acknowledged that the MINI was extensive and covered many disorders. They often expressed that the MINI included their problems: “There were different questions that I liked and that were really good […]. The questions made me feel that they were about me” (Patient 21).

#### The MINI does not evoke negative emotions

According to the questionnaires, few patients felt interrogated during the assessment and few found it exhausting. Similarly, the interviewers seldom felt emotionally exhausted after conducting the assessment.

These negative aspects were not raised spontaneously by the patients in the interviews. After probing, the patients mostly expressed that they were not bothered by the interview. This finding was corroborated by the interviewers, who deemed that the MINI was neither provoking nor offensive to the patients. However, the patients also expressed that it could be difficult to admit to awkward symptoms.

### Perceived advantages of using the MINI for GPs

#### More accurate diagnoses facilitate the work of the GP

The participants acknowledged that the MINI improved the accuracy of the diagnostic procedure. Patients stated that the MINI ensured that important issues were not missed. An example of this, mentioned by the referring GPs, was that distinguishing between depression and anxiety disorders could be tricky for GPs without a special interest in psychiatry. One therapist perceived that the GPs did not probe for details and that the MINI added information by, e.g., asking specific questions about feeling weak, speaking slower than usual or being restless.

The MINI facilitated open discussion of sensitive or awkward issues. It ensured that problems such as addiction and suicide attempts were included and that symptoms suggestive of, e.g., phobias and obsessive-compulsive disorder (OCD) were communicated by the patient. The MINI could thus uncover problems that were unknown to the health care provider.

“Sometimes the MINI confirms what you had suspected. Sometimes it doesn’t, and that is when you think - what luck that I used the MINI, otherwise I wouldn’t have picked up the problem” (Therapist 3).

GPs with experience conducting the MINI perceived that their assessments became more professional when they used the instrument. Instead of simply asking, “Oh, how are you?” (GP2, PCC2), they thoroughly worked through a standardized questionnaire.

#### A useful standard test for deeper investigation and selected patients

The GPs described various experiences and routines regarding the use of the MINI. It could be perceived as a standard test for psychiatric complaints in parallel with somatic problems:

“The MINI has its place. I think that we (GPs) should be able to diagnose psychiatric disorders and know who should be treated in primary care and who should be referred to psychiatry. Of course, we build considerably on our own experience, for example, for those who pee frequently, we often use tests to check for diabetes. I believe that it is good to have a routine test for psychiatric problems as well” (GP2, PCC2).

The GPs chose the MINI in particular when a deeper investigation was required, for example when symptoms were not clear, or the patient had not responded to selective serotonin reuptake inhibitor (SSRI) treatments as anticipated. The MINI was also viewed as an aid for managing silent patients as well as patients with somatoform disorders.

“The MINI is good for patients with somatoform disorders. I performed the MINI with such a patient. It was OK only because it is so standardized and not focused on just *her* psychiatric problems. I found some border psychotic problems, which I believe I never would had thought of asking myself” (GP1, PCC2).

However, some patients could be reluctant. Patients with known psychiatric problems who had been aware of their diagnosis for a long time, especially the elderly, were less motivated to participate in an extra visit to health care for the interview, as were patients with somatic complaints who did not accept that they could have a psychiatric problem.

The MINI was also useful for referrals to specialist care in psychiatry, with participants stating that the explicit information obtained from the MINI facilitated the referral. However, the belief that the MINI was not necessary for a diagnosis, that anamnesis in combination with a depression or anxiety rating scale was sufficient, was also raised. Another value to GPs was that the MINI helped rule out psychiatric diagnoses. One example was that the MINI could be a quick procedure for patients with chronic back pain. Another example was provided as follows:

“A young girl had been at several hospitals for numbness and had gone through many examinations […]. Then, she came to me, and my clinical instinct said depression as an underlying cause for her problems. But I referred her for the MINI, and it was negative on two occasions. So, the MINI was very good; without it, I would have prescribed an antidepressant…” (GP1, PCC1).

### Perceived advantages of using the MINI for the patients

#### New insight into the problem

The interviewers stated that the patients obtained good insight into their problems. In the questionnaire, patients agreed to a lesser extent. One third acknowledged that they understood their problems better after the MINI, and one out of five felt unclear about the results (Table 4). However, the interviewed patients expressed that the MINI gave them better insight. Patients with a MINI diagnosis understood that they had a psychiatric problem, that there was a true cause underlying their symptoms and that they needed help. The questions helped them reflect on their wellbeing in depth and focus their attention on aspects of their problems. The MINI could also help raise issues that the patients were not aware of. One example was a patient who, during the MINI, realized that she consumed too much alcohol and described it as a “wake-up call” (Patient 1). Additionally, the interviewers specifically mentioned the alcohol module:

“But the largest aha-experience was with alcohol. The first question is general, so you can go on with the other questions. It is really interesting to see how people just sit there and reflect on their drinking habits – ‘what relation do I have to alcohol?’ I think this has been very good for many patients” (Therapist 1).

#### The MINI is mostly meaningful

According to the questionnaire, the MINI had been of value to the patients, and most patients agreed that they felt they had been taken seriously (Table 4). In the interviews, patients often concluded that the MINI had been meaningful for them:

“It was great! I score it eight out of ten” (Patient 21).

Having a diagnosis was perceived as a relief and could also be the starting point for patients to read about their problems and how to address them. However, some patients felt that the interview was irrelevant and that they would have preferred spending the time talking with a psychologist instead. The interviewers mentioned that although some patients might meet the criteria for a diagnosis in the MINI, the symptoms associated with the diagnosis were not of major concern to the patient. Other mental problems such as stress bothered them more. The GPs noted that patients were willing to complete the MINI when the purpose was explained to them. Patients expressed afterwards that they were satisfied with the thorough procedure.

#### The MINI may lead to better treatment

Patients, interviewers and GPs expressed that the MINI could have an impact on treatment. Patients who were not satisfied with their current treatment hoped that the results of the MINI would support changing it. The GPs appreciated that the MINI could help them determine the appropriate treatment. One example of this was a patient with sleeping problems who was seeking a renewed prescription for sleeping pills. The MINI showed that the patient had severe depression. After anti-depressive treatment, the patient no longer needed the sleeping pills.

### Perceived weaknesses of the MINI

#### It is a constraint to only answer yes and no

Both patients and interviewers often perceived that the MINI was constraining, which could result in frustration from the patient. Patients expressed that “yes” and “no” were not sufficient response options. One problem was that sometimes none of the alternatives were considered correct. Another problem was that the patients wanted to explain and report details that they themselves considered important. Some stated that the MINI lacked questions that asked for more information. Patients sometimes felt that the task was impossible and answered with their own words. The perceptions of the patients were shared by the therapists. They felt that it was necessary to complement the MINI with their own questions to obtain more detail:

“I always use the questions (of the MINI) in a structured way, but I also ask follow-up questions such as “can you give an example?” or “OK, what do you mean by that?” Otherwise, I don’t think that the MINI adds that much value” (Therapist 3).

### Some questions are problematic, and some common problems are not covered

Although most questions in the MINI were not problematic, some issues did emerge. Both patients and interviewers perceived that the questions relating to pastimes and to the duration of symptoms were harder to answer than questions relating to the current situation. Some questions were seen as extreme, with patients specifically noting those on suicide and addiction. The interviewers highlighted issues with the questions about suicide (“sneaky”) and compulsive behavior (the examples were not familiar to the patients). Other questions were unclear and difficult to interpret: “The questions were perhaps not laser-sharp; it [the MINI] felt like a work in progress” (Patient 4).

The therapists noted that some problems that were common in primary care were not addressed in the MINI. They would have appreciated if stress, fatigue and sleep had been included in the MINI as well. Likewise, some patients expressed that the MINI was not suitable for everybody’s problems:

“The MINI did not suit me perfectly, for example. It felt like the MINI more looked for and tried to capture bipolar problems, not the more “low-key un-gladness”- the sense of never being glad, I mean” (Patient 17).

### The results of the MINI may be biased

One aspect of the MINI that was mentioned was whether the results were valid. Both interviewers and patients recognized that the results could be biased by interpretation and the extent to which the patient answered truthfully. One out of six patients reported that they had not reported everything that had bothered them, while the interviewers suspected that only one out of 17 patients had not reported everything bothering them.

In the interviews, patients noted that they had to interpret questions that they felt were imprecise. They sometimes perceived that there was no such thing as a clear cut “yes” or “no” answer, and thus they guessed, made a best estimate or responded with their own words instead. The interviewers confirmed that they often had to interpret the answer. This could take the form of a dialogue, in which patients and interviewers decided on the most correct alternative together, or patients could leave the responsibility to the interviewer to translate. The interviewers also expressed that certain words in the MINI such as “ever”, “regularly” and “almost completely” were not “crystal clear” (Therapist 2).

The patients noted that the assessment could be subject to manipulation, although some described the MINI as self-correcting and not sensitive to influence. Patients emphasized that they had to be honest if they wanted to receive adequate treatment. However, some confessed that they recognized the diagnoses behind the various modules:

“I was rather aware of how I was. I had googled a little and read about anxiety and other problems. So, I understood what the questions were aiming at, I understood that now, [this question] is about depression, now about anxiety… so it was fairly easy to see through [the intent]” (Patient 19).

#### The duration of the MINI as a potential concern

The recorded mean duration of the 117 MINI interviews was 26 (SD 11) minutes, ranging from 12 to 60 min. More than half of the interviews (54%) lasted 20 min or less, 29% between 21 and 30 min, and 17% between 31 and 60 min.

### The duration was acceptable to the patients

The time required to perform the MINI was seldom brought up by the interviewees. Some patients, after probing, noted that the MINI was time-consuming, but as one patient stated, “… as it helped me so much, it would have been worth even more time” (Patient 11).

#### The MINI most often takes a short period of time according to the interviewers

The interviewers, therapists and GPs found that the MINI was most often a rapid procedure, as patients in primary care generally have a limited range of psychiatric problems. However, patients who quickly lost the thread of conversation or had cognitive difficulties could require more time.

#### It could be problematic to fit the MINI into the GP consultation scheme

Some GPs who conducted the MINI were wary of the time needed. Even if the assessment took only 20 min, they often had to book a separate consultation for the MINI. This could be weeks later, which was not acceptable for patients with severe problems. These GPs would have appreciated a shorter or web-based version of the MINI in order to save time. A wish to use only sections of the MINI that were perceived to be relevant to the actual patient was also expressed.

### The MINI is an additional tool and a personal contact with the interviewer is important

#### The MINI is just one part of the diagnostic procedure and its role must be explained

Patients were not always clear about the role of the MINI in the diagnostic procedure. Some patients seemed to believe that the purpose of the MINI was to replace the clinical assessment. They stressed that, on principle, the MINI could not be sufficient for a diagnosis but must be combined with a conversation.

For the interviewers and GPs, the MINI could be more or less important for the final diagnosis. Some interviewers weighed the patient’s story, the patient’s behavior and the results of the MINI. If contradictory, the patient’s behavior and story were more important for deciding how to manage the patient than the MINI diagnosis. Others based the final diagnosis on the MINI only but found that a short discussion after the interview could result in valuable additional information. The referring GPs found that the MINI provided useful extra information, but they emphasized that their own judgement was the most important.

Some patients found that the information and instructions for completing the MINI were not sufficient. Written instructions to read in advance or short outlines of the structure of the MINI before the interview would have been helpful. Insufficient information could have unintended consequences. Not understanding the reason for the interview could lead patients to feel uncertain about how the results would be used. Patients who did not obtain a diagnosis could perceive that the interview was irrelevant. The interviewers added that thorough instructions were vital for patients to adhere to the “yes-no” structure.

#### An interpersonal contact with the interviewer is important

In the questionnaires, patients as well as interviewers expressed that they had established a positive relationship during the interview. In the interviews, the importance of interpersonal contact was expressed by both patients and interviewers:

“If I had come to the physician and felt pretty rotten and had then been interviewed by a counselor or whatever … and that person just read from a template, then maybe, I would think, ‘Please, I need help, and you are just reading from a checklist’. I could imagine that” (Patient 22).

Alternatively, a therapist noted the following:

“Some (patients) have kind of felt that they did not have the opportunity to talk (…) So it is a question of weighing up how much [to use the] instruments… there must also be time to tell in your own words how you feel. You should not underrate personal meetings” (Therapist 1).

However, for some patients, it was viewed as an advantage that there was no need for a personal relationship with the interviewer. One perception, expressed as positive, was that no eye contact was required and that the MINI “became like armor” (Patient 3) that prevented personal boundaries from being crossed.

## Discussion

This study described the experiences and perceptions of the use of a psychiatric structured interview instrument, the MINI, in primary care. The MINI was considered a useful part of the clinical assessment of selected patients with unclear psychiatric symptoms. Furthermore, the MINI helped GPs establish an accurate diagnosis as the basis for choosing treatment options. Patients were satisfied with the MINI, which helped them understand and verbalize their problems. The MINI was not emotionally disturbing for the patients. The yes-no format could be a constraint, and subjective interpretations were possible. Lack of time was a concern only when the MINI was performed by GPs.

Patients rated the MINI as a relevant procedure. Many of them expressed that they preferred to answer standardized questions, as it was difficult for them to tell their story in their own words. The MINI helped them communicate their problems. They could identify parts of themselves in the questions, which also facilitated the disclosure of shameful or embarrassing problems that they otherwise would not have shared. Although some questions might have been disturbing, very few patients found the MINI to be intrusive or problematic. Patients’ perceptions of psychiatric structured interviews in primary care have not been reported previously, but our results correspond to what has been described in studies from other settings, mostly in psychiatry [23, 24, 28, 32, 33]. However, it should be noted that approximately 3% of the patients found the procedure unnecessary and would have preferred to have more time for a less structured conversation.

The referring GPs and interviewers in our study perceived that the major advantage of the MINI was that it contributed to an accurate diagnosis for patients who needed a more thorough investigation and that it helped identify comorbidities. A third of the patients presented with comorbidities, mostly depression accompanied by one or more anxiety disorders. A substantial proportion, one out of ten, had other comorbidities such as bipolar disorder, OCD, Post-Traumatic Stress Disorder, suicidal behavior or alcohol dependence, conditions that were not previously known to the GPs. These figures may, however, be an underestimate. The two therapists employed at PCC2 differed from the other interviewers regarding number and type of diagnoses. They mostly reported one diagnosis only, whereas the others were more likely to report all diagnoses indicated by the MINI. One explanation of this finding could be that the therapists at PCC2 only reported the diagnosis that was the focus of their treatment.

The GPs acknowledged that an accurate diagnosis affected the choice of treatment. One option for Swedish primary care physicians when managing patients with suspected depression or anxiety is to test whether the patient improves with selective serotonin reuptake inhibitor (SSRI) treatment. As one out of five in our study did not meet the criteria for depression or anxiety disorder, this indicates that the MINI could help decrease unnecessary treatment and shorten the duration of the search for correct management. This is in accordance with another study, which found that a proportion of patients, who had received a diagnosis of affective or anxiety disorders based on clinical parameters, were subthreshold cases, not needing medical treatment [12]. Knowledge of hidden comorbidities also affects treatment and may lead to more tailored treatment or referral to psychiatry. Psychiatric disorders that are perceived as stigmatizing to the patients are less often identified by conversations with the patient than when the conversation is supplemented with a structured interview [34].

The GPs in our study concluded that the MINI was a useful addition, especially for complicated patients, which is in line with a previously mentioned study in Brazil [22]. However, it should be underscored that the MINI, like other structured interviews, is limited to specific psychiatric diagnoses and does not cover all mental problems encountered in primary care [35]. This was also observed by patients and interviewers in our study, as some of them missed modules about stress and sadness.

We have not found any other studies that investigated GPs’ experiences of using a structured interview for mental illness. However, our results are more positive than those shown in other studies on the acceptance of short, self-rating questionnaires for case-finding and severity of depression. Such tests have for example been studied as part of the introduction of the guidelines from the National Institute for Clinical Excellence (NICE) [36]. Some studies highlighted a concern that the doctor-patient relationship could be compromised, in particular that good conversations might be disturbed by introducing a document that should be followed [37–41]. This aspect was not mentioned in the present study, as GPs either had chosen to work with the MINI themselves or did not face this situation because the patients were referred to a psychologist for the MINI.

Although advantages of the MINI for patients as well as for GPs were highlighted, some other aspects should be taken into consideration. Patients and interviewers often found that the yes-no format was limiting and developed ways to communicate more information. For example, patients could refuse to answer yes or no or could talk freely and leave it to the interviewer to translate their response. Some interviewers with more experience added open-ended questions. Thus, in practice, the MINI was sometimes used as a semi-structured interview. This is acceptable according to the instructions of the MINI but requires greater skills and more in-depth training of the interviewer.

A related issue, raised by patients and interviewers, concerned the validity of the MINI. Apart from the risks of misunderstanding during the interpretation of questions and answers, patients may want the interview to have a certain outcome. Interestingly, the interviewers seldom suspected that the patients had withheld information, although a sixth of the patients reported falsely negating problems assessed in the MINI. Despite these omissions, the patients seemed to be aware that truthful answers were important for their treatment. However, regardless of the approach used to diagnose psychiatric disorders, the diagnosis is solely based on information provided by the patient and/or their relatives. During consultations, information about psychiatric problems and the interpretation of this information have been shown to be dependent on certain factors, e.g., patient’s gender, marital status and quality of life [12], non-verbal communication [42] and GPs’ prior knowledge of the patient’s other problems and contextual factors [8]. Thus, the risk of distorted information is general and not unique to the MINI.

The time required for assessment is an important aspect of tests performed in primary care settings. In our study, the average duration of the MINI was 26 min, which is in line with the times reported in other studies [22, 26]. Time was not viewed as a major barrier to the use of the MINI. However, most interviews were performed by therapists, whose time was less restricted. Some GPs who conducted the MINI, on the other hand, perceived that the consultation time could be too short for completing the MINI. This finding is in accordance with the results of a Brazilian study [22] in which GPs used the MINI. In that study, the duration of the MINI was considered an obstacle to its use as a screening instrument but was considered acceptable for use with selected patients.

### Strengths and limitations

Our study is the first to explore primary care patients’ perspectives toward the use of a structured interview for mental disorders. A strength of the study was the design, which addressed the implementation of the MINI from multiple perspectives. The sample was broad, with three participating PCCs from areas with varying socioeconomic statuses, GPs who had little to substantial experience, and patients with varying characteristics with respect to gender, age, ethnicity and mental problems. Furthermore, experiences and perceptions were obtained through questionnaires that were supplemented with richer information from semi-structured interviews and focus groups. The triangulation between participant groups and data collection methods showed consistent results. The content analysis was conducted by two researchers with different backgrounds and preconceptions, which allowed for different perspectives and reflexivity [43]. It should be noted that the GP at PCC3, who was also a co-author of this publication, had no access to the primary data and did not take part in the analysis.

The study protocol stated that 10–12 patient interviews should be conducted. This was a practical consideration that was made in order to avoid obtaining an amount of information that might be difficult to grasp. As suggested by Malterud et al. [44], the sample size was reevaluated. Our preliminary analysis showed that several of the interviews resulted in a limited amount of information and that more interviews were needed to more fully capture the variations in perceptions. After another 12 interviews had been conducted, the data were broader, and the final interviews did not yield any new information.

The sampling method may have led to limitations in the transferability of the results. Owing to the lack of financial support, few PCCs felt inclined to participate, although key politicians and the primary care board deemed the project important for practice. The PCCs that participated were interested enough to invest in the study. Better management of patients with mental ill-health and thus better diagnostic procedures were important for these PCCs. The experiences and perceptions of interviewers and GPs may differ in PCCs with other values and priorities, although patient perceptions are likely more stable. Another factor that may affect transferability is that the experiences were confined to the paper-and-pen version of the MINI 6.0. Changing from the MINI 6.0 to the recently introduced MINI 7.0 would probably not influence these perceptions. However, the web version of the MINI may lead to other perceptions of the procedure from both the patients and the interviewers.

Unexpectedly, the commitment to the study differed between the sites and over time. Two PCCs had a local study coordinator who ensured that the procedures were followed. The third PCC underwent a reorganization when the study coordinator left, and the personnel had to focus on implementing new routines. This affected the data collection, which became less systematic. One of the employed therapists did not complete ten questionnaires and time recordings because of time constraints. However, there was little variability in the existing 115 questionnaires, and there is no reason to believe that the missing questionnaires would have changed the results substantially.

The credibility of the findings may be affected by time. The study was conducted during one full year, and there might be a risk that the experiences changed as a result of internal or external events, such as the reorganization of PCC2. However, the interviews and questionnaires did not indicate such a shift over time. On the other hand, during the analysis, it became apparent that some patients had changed their perspectives from the time when the questionnaire was completed to the research interview, which was conducted several weeks later. At the interview, the patients had most often been informed of their diagnosis, had started treatment, and had also had time for reflection. However, there was no clear direction to any of the changes, as patients expressed higher as well as lower acceptance at the interview.

## Conclusions

The paper-and-pen version of the MINI can be useful in primary care as part of the clinical assessment of patients at risk of depression and anxiety. The MINI helped to obtain a complete picture and to identify psychiatric comorbidities, including stigmatizing disorders. Patients, GPs and therapists appreciated the MINI. It facilitated the GPs’ work, provided new insight for patients and was not experienced as intrusive or exhausting.

## Additional files

Additional file 1: Questionnaires and topic guides. (PDF 203 kb)

Additional file 2: An example of the coding structure. (PDF 263 kb)

## Abbreviations

GP: General practitioner
IQR: Interquartile range
MINI: Mini-International Neuropsychiatric Interview
PCC: Primary care center
VAS: Visual Analog Scale
