# Supplementary material for: The Mini-International Neuropsychiatric Interview is useful and well accepted as part of the clinical assessment for depression and anxiety in primary care: a mixed-methods study
Source: BMC Fam Pract. 2018 Jan 24;19:19. doi: 10.1186/s12875-017-0674-5 (PMC5781342; doi:10.1186/s12875-017-0674-5)
Supplement: Supplementary file 1 — Questionnaires and topic guides. (PDF 203 kb) [file 12875_2017_674_MOESM1_ESM.pdf]

Additional File 1. Questionnaires and topic guides

Table 5. Items in the patient and interviewer acceptance questionnaires [8]

**Patients**	**Interviewers**
1. the procedure used by the interviewer was helpful	1. I conducted the interview as well as I could
2. I feel more confused than before the interview	2. I found it difficult to get all the relevant information needed to clarify the diagnostic picture
3. I felt interrogated	3. While conducting the interview I felt competent
4. there were too many questions	4. I made mistakes in administering the interview
5. the interview was exhausting	5. the patient perceives himself/herself and his/her problems in a differentiated manner
6. I think that the interviewer took my problems seriously	6. the relationship to the patient was positive
7. the relationship to the interviewer was positive	7. I think that the patient did not report everything that was bothering him/her
8. I didn't report everything that was bothering me	8. the interview was exhausting
9. after the interview I have a better understanding of myself and my problems	9. during the interview I experienced the patient as cooperative
10. I think that the interviewer asked for enough detail to get an appropriate understanding of my situation	10. I succeeded in being responsive to the patient

Table 6. Topic guides for the interviews and focus groups discussions about use of MINI in primary care (probing questions in italics)

Interview with patients

What happened when you met with the GP?

Why did the GP refer you to a psychologist?

How did you feel about being referred for an assessment?

Did the referral affect you confidence in the GP? How?

How has your relationship with your GP been affected by the referral?

What were your experiences from visiting the psychologist?

What experiences did you have of the psychologist reading aloud from a questionnaire with fixed yes- and no- responses?

Did you experience any problems, answering the questions? Please describe

Did you recognize yourself in any of the questions? Please describe

When you met with the GP again, what happened?

Did the GP refer to the result of the MINI-assessment? Please describe.

Did you perceive that the GP had understood you and your problems? Please describe.

Do you give the same information to the GP and the psychologist? What are the differences?*

What pros' and cons' did you see with the referral?

Focus group discussions with GPs**

Discuss about experiences from the referral

Which patients were referred? And which were not? Why?

How were the patients' attitudes towards being referred? How do you perceive that the referral changed their confidence in you (capture both relationship and competence)?

Discuss about experiences of using the results from MINI

How did you use the feedback from the psychologist?*

What do you think about the accuracy of MINI?

Discuss the values of using MINI for GPs and patients

What pros' and cons' do you see using MINI as part of diagnosis in primary care? Would you like to continue using it?

What pros' and cons' do you see referring to a psychologist? Would you like to continue?*

Interview with psychologists

What are your experiences of using MINI?
When do you use MINI?
What works well and what does not work well?
How do you perceive that patients' feel about you using MINI?

What are your experiences with MINI?
How do you perceive the structure of MINI and how does the patient perceive it?
How do you perceive the individual items?
