# Supplementary material for: The Mini-International Neuropsychiatric Interview is useful and well accepted as part of the clinical assessment for depression and anxiety in primary care: a mixed-methods study
Source: BMC Fam Pract. 2018 Jan 24;19:19. doi: 10.1186/s12875-017-0674-5 (PMC5781342; doi:10.1186/s12875-017-0674-5)
Supplement: Supplementary file 2 — An example of the coding structure. (PDF 263 kb) [file 12875_2017_674_MOESM2_ESM.pdf]

Additional file 2.

An example of the coding structure; Main category from the patient analysis," the MINI increases the accuracy of the diagnosis and facilitates for the GP"

Meaning bearing unit (Patient ID)	Condensed meaning bearing unit	Code	Subcategory	Category
And then of course the treatment cannot follow a form but for narrowing down I think it is excellent" (P6)	The interview is excellent for narrowing down the problem	Excellent for narrowing down problems	Helps to narrow down problems	MINI makes the problems clear to the GPs
I perceived that the GP got a good overall picture of what I felt although MINI had no questions fitting to my problems. Her conclusions were close enough (P17).	The GP got a good overall picture of my problems even if no questions fitted perfectly.	The GP got a good overall picture of the problems	Gives the GP a good overall picture of the problems	
And… then the doctor could see it clearer (P11)	Makes the doctor see clearer	The doctor sees clearer	The doctor sees clearer	
…otherwise it can be difficult to find a diagnosis because it may be a little of everything and then you as a doctor has to try to arrive at a concrete diagnosis. I believe it is very difficult. Here you have some help on the way with items and questions (P16)	It may be difficult to find the diagnosis because there is a little of everything. Here the doctor gets help with items and questions to arrive at a diagnosis	The doctor gets help to arrive at a diagnosis with items and questions	items and questions facilitate finding the diagnosis	Support to find a diagnosis
When you just report on your own there sometimes are things that you forget and don´t think of. Things were brought up more easily, when these questions were asked about everything (P11)	If you only narrate your story you may forget some things. It came more easily with questions in the interview.	Questions helped bring up things that might otherwise be forgotten	What patients forget to tell, come up with questions in the interview	The questions help not to miss information

..as well as for the doctors…to not miss so much, considering that if someone is telling his/her story he/she may not tell about an addiction or whatever.. it is not withholding so to speak (P18)	The doctor does not miss so much. For example, if someone tells his/hers story and doesn´t bring up that he or she has an addiction. It becomes not withholding.	The doctor does not miss for example addictions that the patient does not admit deliberately.	What patients don't tell deliberately can show up with the interview	
As a doctor…you cannot know everything, in some way you must be able to talk with the patients about anything, and this kind of mental problems must be very difficult to, well handle on your own. To have standardized forms for it I think is a good help actually (P17)	The doctors cannot know everything, and it must be very difficult to talk with the patients about mental problems. Then, I think; a standardized form is a good help for them.	Standardized forms are a good help for the doctors to talk about mental problems with the patients	Standardized questions a good help to talk with the patients	Facilitates the work of the doctor
And then I think it may be a help for the doctors as well. It always is so stressful, and the doctors don't have the time to be empathic, they see so many patients. They do not have the time to understand how a patient feels- and at the same time the patients may not be in the mood to tell, […] If you have it on paper instead the doctors can see " so, this is how you feel". I think that was great. (P3)	The interview can be a help for the stressed doctors as well. They don't have the time to be compassionate and patients may not be in the mood to talk. If you have a paper on how you feel it helps	It facilitates for the stressed doctor when there is a paper on how the patient feels	It facilitates for the stressed doctor when there is a paper on how the patient feels	
